# Continuous planting American ginseng (*Panax quinquefolius* L.) caused soil acidification and bacterial and fungal communities’ changes

**DOI:** 10.1186/s12866-024-03616-7

**Published:** 2024-11-13

**Authors:** Shaoyong Li, Haiwei Wei, Daqian Qi, Wenwen Li, Yan Dong, Fu-Ang Duan, Shou-Qing Ni

**Affiliations:** 1 The Second Prospecting Team of Shandong Coal Geology Bureau, Jining, Shandong 272000 China; 2 School of Environmental Science and Engineering, Shandong University, Qingdao, Shandong 266237 China; 3 The Fifth Prospecting Team of Shandong Coal Geology Bureau, Jinan, Shandong 250215 China; 4 Physical Exploration and Survey Team of Shandong Coal Geology Bureau, Jinan, Shandong 250215 China; 5 Shenyang Academy of Environmental Sciences, Shenyang, Liaoning 110167 China

**Keywords:** Continuous cropping, American ginseng, Soil microbial communities, Diversity, Pathogens

## Abstract

**Background:**

American ginseng is an important herb crop and is widely cultivated in China. However, continuous cropping seriously affects the production of American ginseng, and the reason is still unclear and needs more research. We analyzed the soil microbial alpha diversity and community composition as well as soil physicochemical properties in bulk soils to assess the changes in soil associated with planting American ginseng.

**Results:**

The cultivation of American ginseng resulted in a significant decrease in soil pH value. The alpha diversity of soil bacteria and fungi was significantly reduced with the increase of American ginseng planting years. Planting American ginseng also largely altered the community composition of soil bacteria and fungi, in particularly, increased the relative abundance of the pathogenic fungus *Fusarium*, and reduced the relative abundance of some beneficial microorganisms, such as *KD4-96*, *RB41* and *Sphingomonas*.

**Conclusions:**

Soil acidification, reduction of beneficial taxa and accumulation of fungal pathogens, therefore, may lead to the replantation problem of American ginseng.

## Introduction

American ginseng (*Panax quinquefolius L*.), is a perennial herb, which has been widely cultivated in China with a total cultivation area of nearly 3,700 hectares [1]. Due to its anti-inflammatory and antioxidant properties, it is considered to have good therapeutic effects in enhancing central nervous system function, protecting cardiovascular system and improving immunity [2–4]. As an important herbal medicine, the global market of American ginseng reached 85 million US dollars in 2018, and it is estimated that it will reach 7,033 tons in 2019 to 2024 [5]. China is the third largest producer of American ginseng, producing 3,600 tons in 2016, and is the largest national consumer of American ginseng [1]. American ginseng grows slowly and usually needs for more than 4 years to achieve optimal medicinal value [6]. However, long-term monoculture can cause obstacles in continuous cropping, mainly manifested as the increase of soil-borne diseases and the significant reduction of crop yield [7]. Therefore, overcoming the continuous cropping obstacle is important for the sustainable development of American ginseng.

Continuous cropping obstacles are a common but difficult problem in agriculture, which affect many crops, such as apple, strawberry, alfalfa and grapevine and result in severe losses of crop yield [7–9]. In China, continuous cropping obstacles have become a major factor restricting the planting area growth of American ginseng [10, 11]. Moreover, it is estimated that successful replanting needs a crop rotation cycle of at least 30 years [12], which means the land suitable for American ginseng cultivation in China is rapidly decreasing [1]. Replanting problems are generally caused by multiple factors, including the changes of soil properties, autotoxicity, outbreak of soil diseases, and changes in soil microbial communities [10, 13, 14]. Most of researches have focused on allelopathy/autotoxicity, soil nutrient element depletion or soil sickness [15, 16]. For examples, it was found that 9 phenolic compounds identified from fibrous roots of American ginseng significantly inhibited the growth of seedlings [17]. Bi et al. [15] have reported 9 phenolic compounds that can inhibited the radicle and shoot growth of American ginseng. Additionally, disturbance of soil microbial communities is also closely related to continuous cropping obstacles. Soil microbial communities, involved in almost all biogeochemical cycles of terrestrial ecosystems, play an important role in agricultural production, and are irreplaceable in maintaining a variety of ecosystem functions and services, such as nutrient cycling, vegetation primary productivity and litter decomposition [18]. There are some studies have explored the relationship between continuous cropping and soil microorganisms [1, 5, 19]. These studies have shown that changes in the diversity of soil microbial communities contributed to continuous cropping obstacles of American ginseng. However, these studies mainly focus on Northwest and Northeast China. The investigation about continuous cropping of American ginseng in North China are limited, where the climatic conditions are obviously different from the Northwest and Northeast China. Therefore, it is necessary to carry out relevant research in North China to provide targeted theoretical support for the cultivation of American ginseng in different areas.

Therefore, in this study, from the perspective of soil microbial communities, we examined the changes in the alpha diversity of fungi and bacteria, the bacterial and fungal community and functional composition in the soil from American ginseng farmland in Shandong Province, which is main producing area of American ginseng in China. American ginseng in this study has been planted for different years (from 0 year to 4 years). This study aimed to find the main factors associated with replanting problems and provided theoretical basis for finding new strategies to solve continuous cropping obstacles.

## Materials and methods

### Experimental design and sample collection

In November 2020, the soil samples were collected in Wendeng District, Weihai City, Shandong Province (121°43’E-122°19’E, 36°52’N -37°22’ N), one of the main planting areas for American ginseng in China. One crop season of American ginseng usually needs 4 years. To reduce geological variability, soil samples were random collected from 15 sites nearby in the research area, where American ginseng had been planted for 0–4 years respectively (Y0, Y1, Y2, Y3 and Y4). Y0 were set as the control, where American ginseng had never been planted. There were three replicates for each growth year, resulting in a total of 15 samples. Using five-point sampling method, five soil cores (20 cm deep, 5 cm diameter) were collected from each site by a soil auger. Soil from the five cores were mixed and sieved through a 2 mm mesh.

Soil pH was measured in 1:2.5 (soil: water) water suspensions by a pH meter (310P-02 A, Thermo Fisher Scientific, America). The flame photometric method was used to determine soil available potassium (M410, Sherwood Scientific Ltd., Cambridge, UK). Soil inorganic N (NH_4_^+^ and NO_3_^−^) was extracted from 10 g (wet mass) fresh soil with 50 ml of 2 mol/L KCl, and then determined using an auto flow injection analyzer (AA3, Bran + Luebbe, Germany).

### Soil DNA extraction, PCR amplification and sequencing

For each soil sample, soil microbial genomic DNA was extracted from 0.5 g of mixed soil using the MP FastDNA SPIN Kit for soil according to the manufacturer’s instructions (MP Biochemicals LLC, Solon, OH, USA). We quantified the quality and concentration of the extracted through the NanoDrop ND-2000 Spectrophotometer DNA based on 260/230 nm and 260/280 nm absorbance ratios (NanoDrop Technologies Inc., Wilmington, DE, USA). The amplicons of the bacterial V4 hypervariable regions of the 16 S rRNA gene and fungal ITS1 region of the 18 S rRNA gene were sequenced to test the soil bacterial and fungal diversity and composition. The primers 515 F (5′-GTGCCAGCMGCCGCGG-3′) and 907R (5′-CCGTCAATTCMTTTRAGTTT-3′) were implemented to amplify the V4 hypervariable regions of the 16 S rRNA gene. The ITS1 region of the fungal 18 S rRNA gene were amplified using primers 2043R (5′-GCTGCGTTCTTCATCGATGC-3′) and ITS1F (5′-GTGCCAGCMGCCGCGG-3′). All the PCR reactions were performed in 30 µL reactions containing 0.2 µM of forward and reverse primers, 15 µL of Phusion^®^ High-Fidelity PCR Master Mix (New England Biolabs) and about 10 ng of template DNA. The PCR conditions used were as follows: 98 ºC for 1 min, 30 cycles of denaturation at 98 ºC for 10 s, 50 ºC for 30 s, and 72 ºC for 30 s, finally extension 72 ºC for 5 min. The amplicons were mixed together with the equal volume of loading buffer and detected in a 2% agarose gel by electrophoresis. Equimolar amounts of amplicons were then purified by a Gel Extraction Kit (Thermo Scientific). The Illumina TruSeq DNA PCR-Free Library Preparation Kit (Illumina, USA) was used for generating the sequencing libraries. The Agilent Bioanalyzer 2100 system and Qubit@ 2.0 Fluorometer (Thermo Scientific) were used to assessed the library quality. finally, the library was sequenced through an Illumina HiSeq platform and 250 bp paired-end reads were obtained.

### Sequence data processing and statistical analysis

The paired-end reads were merged and quality filtered by FLASH (V1.2.7) [20] and QIIME (v1.7.0) [21]. The chimeric sequences were removed using USEARCH software [22]. After removing the chimeric sequences, the OTUs (Operational Taxonomic Units) were clustered at 97% similarity by Uparse (v7.0.1001) [23]. The Silva database [24] for bacteria and Unite database [25] for fungi were used to assign each OTU to taxonomic group. The sequence numbers of the sample with lowest number of reads (18742 reads for bacteria and 35051 reads for fungi) were used to rarify each sample. The QIIME (V1.7.0) [21] were used to calumniate the alpha-diversity indexes (OTU richness, Shannon diversity and the Chao1 index).

Metabolic potential of functional genes were predicted by the Phylogenetic Investigation of Communities by Reconstruction of Unobserved States (PICRUSt2). The 16 S rRNA gene and 18 S rRNA gene amplicon sequencing data were against to MetaCyc Metabolic Pathway Database (https://metacyc.org/) with known metabolic functions to obtain the prediction of bacterial and fungal metabolic function.

General linear or quadratic model regressions were performed to evaluate the relationship between the growth years of American ginseng and the soil physicochemical properties (soil pH, soil available potassium, soil inorganic N), soil microbial alpha diversity indices, the relative abundance of dominant (Top 10) bacterial phyla and fungal classes, as well as the relative abundance of bacterial and fungal functional guilds. These data analyses were performed with OriginPro (v 9.0, Massachusetts, USA). The effects of growth years (Y) on the bacterial and fungal community composition were tested using PERMANOVA (Permutational Multivariate Analysis of Variance) with 999 permutations. To show the relative difference of bacterial and fungal communities and functional composition in different years, we used the NMDS (Non-metric Multidimensional Scaling) plots based on the Bray-Curtis dissimilarity matrix. The RDA (Redundancy Analysis) was further performed to test the effects of soil physicochemical properties on bacterial and fungal communities and functional composition. These statistical analyses were performed in R (V3.5.1; R Core Team. 2013) with vegan package [26].

## Results

### Soil physicochemical properties

**Fig. 1:**
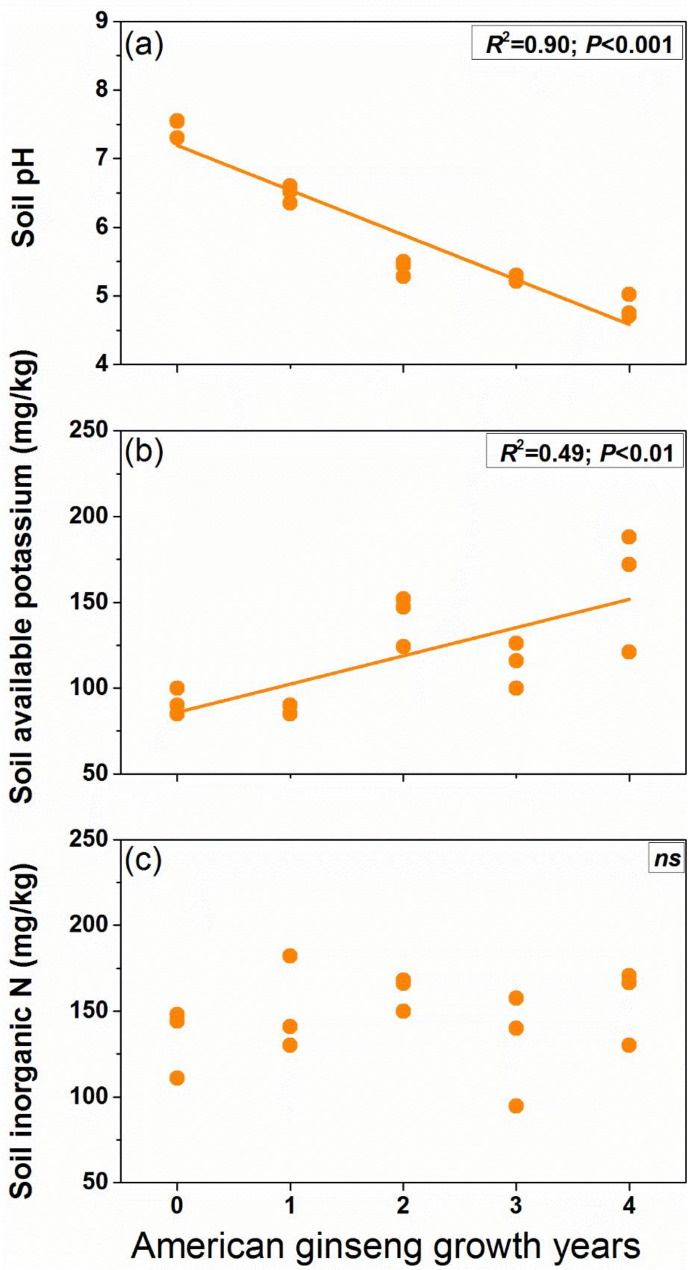
The relationship between Soil properties and American ginseng growth years. For each response variable, regression was estimated using a linear model with years of American ginseng cultivation as a continuous predictor. *R*^*2*^ is coefficient of determination; *P* is significance of the regression, *ns* means not significant

The cultivation of American ginseng resulted in a significant decrease in soil pH value. The soil pH was negatively correlated with the growth years of American ginseng (*R*^*2*^ = 0.90; *P* < 0.001; Fig. 1a). In the control fields without American ginseng planting, the soil pH was about 7.46, however, after four years, it dropped to about 4.83. On the other hand, the content of soil available potassium showed positively correlated with the American ginseng growth years and increased with the years of American ginseng cultivated (*R*^*2*^ = 0.49; *P* < 0.01; Fig. 1b). There was no significant correlation between soil inorganic N and American ginseng growth years (Fig. 1c).

### Bacterial and fungal alpha diversity

**Fig. 2:**
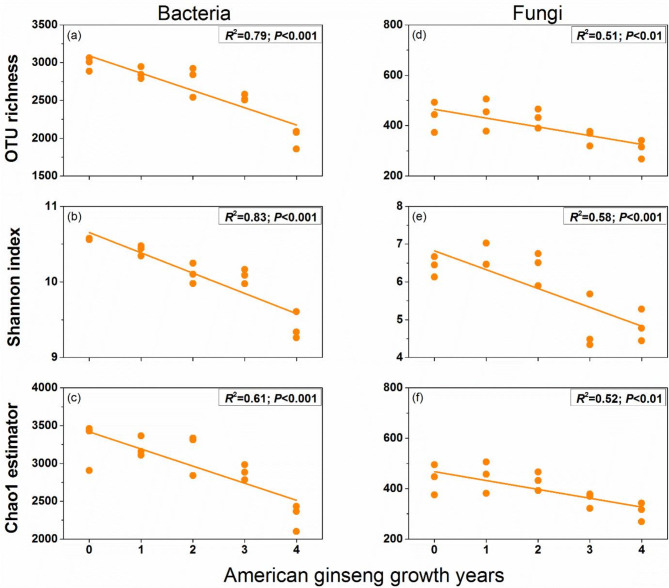
The relationship between soil bacterial and fungal alpha diversity indices and American ginseng growth years. For each response variable, regression was estimated using a linear model with years of American ginseng cultivation as a continuous predictor. *R*^*2*^ is coefficient of determination; *P* is significance of the regression

Planting American ginseng had a strong effect on soil microbial alpha diversity (including OTU richness, Shannon index and Chao1 estimator). For bacterial communities, the alpha diversity index showed an obvious linear correlation with the growth years of American ginseng. The OTU richness (*R*^*2*^ = 0.79; *P* < 0.001; Fig. 2a), Shannon index (*R*^*2*^ = 0.83; *P* < 0.001; Fig. 2b) and Chao1 estimator (*R*^*2*^ = 0.61; *P* < 0.001; Fig. 2c) of soil bacteria significantly decreased with the increase of the growth years of American ginseng. Similarly, for fungi, the OTU richness (*R*^*2*^ = 0.51; *P* < 0.01; Fig. 2d), Shannon index (*R*^*2*^ = 0.58; *P* < 0.001; Fig. 2e) and Chao1 estimator (*R*^*2*^ = 0.52; *P* < 0.01; Fig. 2f) declined with the growth years of American ginseng.

Furthermore, results from a linear regression analysis revealed a remarkable positive correlation between soil pH and the alpha diversity of bacteria and fungi, while soil available potassium was negatively correlated with the alpha diversity of bacteria and fungi (except the Shannon index of fungi). There was no significant correlation between soil inorganic N and the alpha diversity of bacteria and fungi (Table 1).

**Table 1:** Relationship between soil pH, soil available potassium, soil inorganic N and alpha diversity indices from a linear regression analysis

		Soil pH	Soil available potassium	Soil inorganic *N*
Bacteria	OTU Richness	0.78***	-0.72**	-0.16
	Shannon	0.85***	-0.83***	-0.24
	Chao1	0.65**	-0.65*	-0.08
Fungi	OTU Richness	0.64**	-0.56*	0.05
	Shannon	0.67**	-0.49	-0.01
	Chao1	0.65**	-0.57*	0.05

The correlation coefficients (Pearson’s *r*) are presented with the level of significance. * *P* < 0.05; ** *P* < 0.01; *** *P* < 0.001

### Bacterial and fungal communities and functional composition

**Fig. 3:**
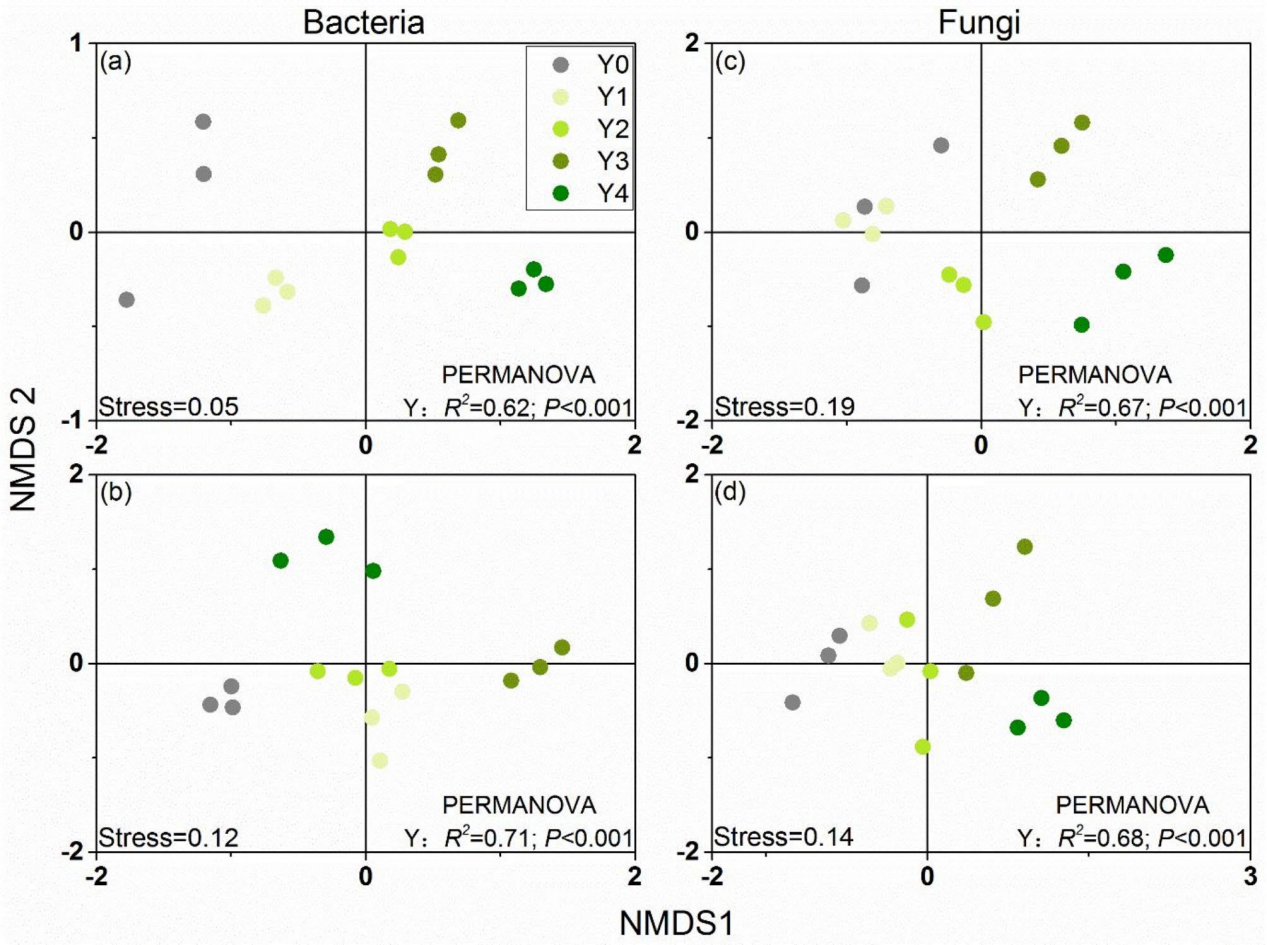
Nonmetric multidimensional scaling (NMDS) ordination of soil bacterial community composition (**a**), functional composition (**b**), fungal community composition (**c**) and functional composition (**d**). Results (*R*^*2*^ and *P*-value) of multivariate permutational analysis of variance (PERMANOVA) on the effects of years of American ginseng cultivation (Y) also shown

The NMDS plot revealed that the distribution of bacterial and fungal communities and functional composition was clear separated by years of American ginseng cultivation (Y). The multivariate permutational analysis of variance (PERMANOVA) results also showed that the bacterial and fungal communities and functional composition varied significantly among different planting years and the years of American ginseng cultivation had a high explanation (Fig. 3a-d). These results implied that planting American ginseng significantly changed the community and functional composition of soil bacteria and fungi.

**Fig. 4:**
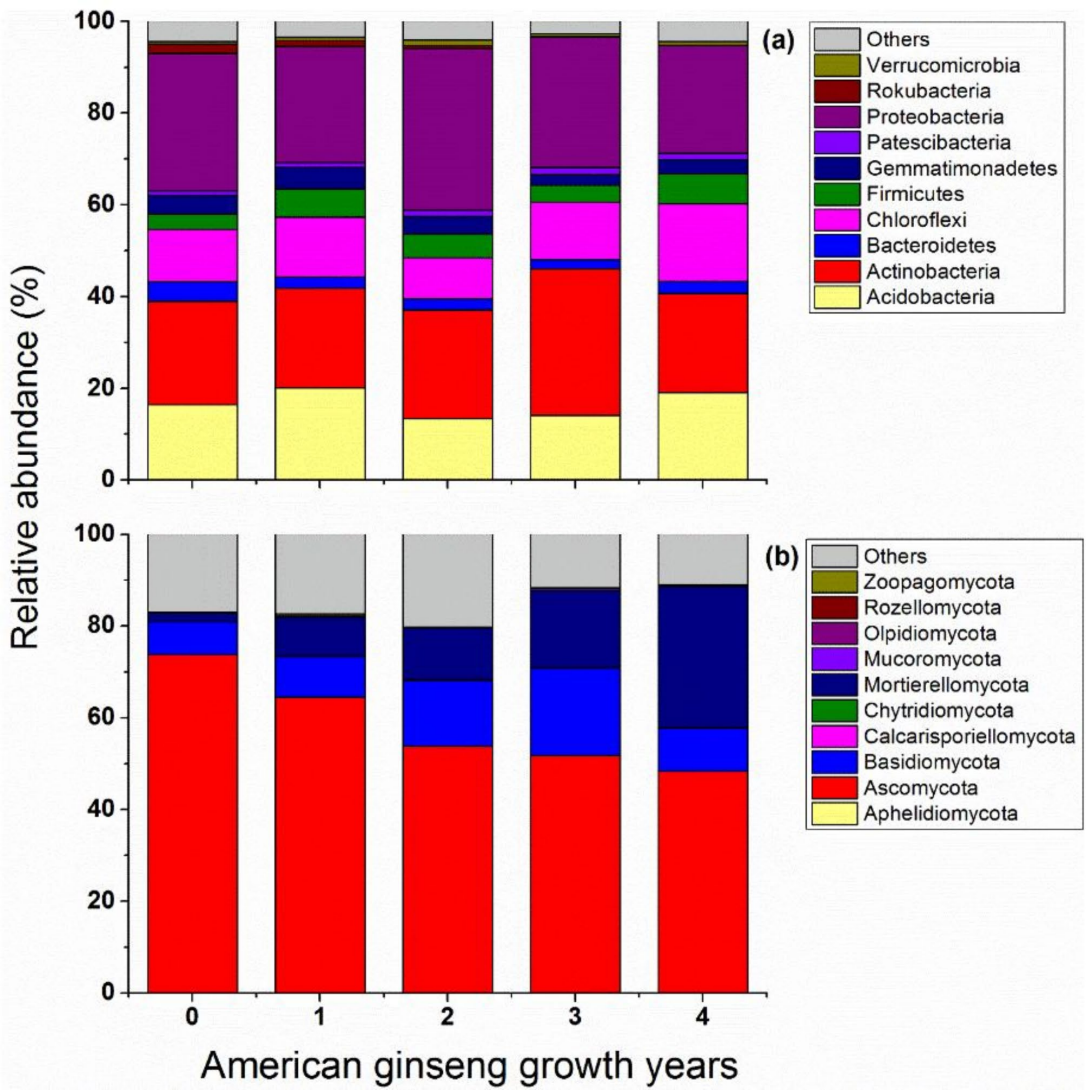
The relative abundance of dominant (top 10) bacterial phyla (**a**) and fungal phyla (**b**) in different American ginseng growth years

Further analysis at different taxa classification levels found that, at the bacterial phyla level, the top 10 dominated phyla were Proteobacteria (23.4-25.3% of total relative abundance), Actinobacteria (21.6-23.7%), Acidobacteria (13.4-20.1%), Chloroflexi (9.0-16.8%), Firmicutes (3.3-6.6%), Gemmatimonadetes (3.0-4.8%), Bacteroidetes (2.3-4.2%), Patescibacteria (1.0-1.4%), Rokubacteria (0.1-2.1%) and Verrucomicrobia (0.6-1.1%) (Fig. 4a). For fungi, the most abundant sequences were dominated by the phylum Ascomycota (48.3-73.8%), while the phyla Mortierellomycota and Basidiomycota represented only 1.9-30% and 7.1-14% reads, respectively (Fig. 4b).

**Fig. 5:**
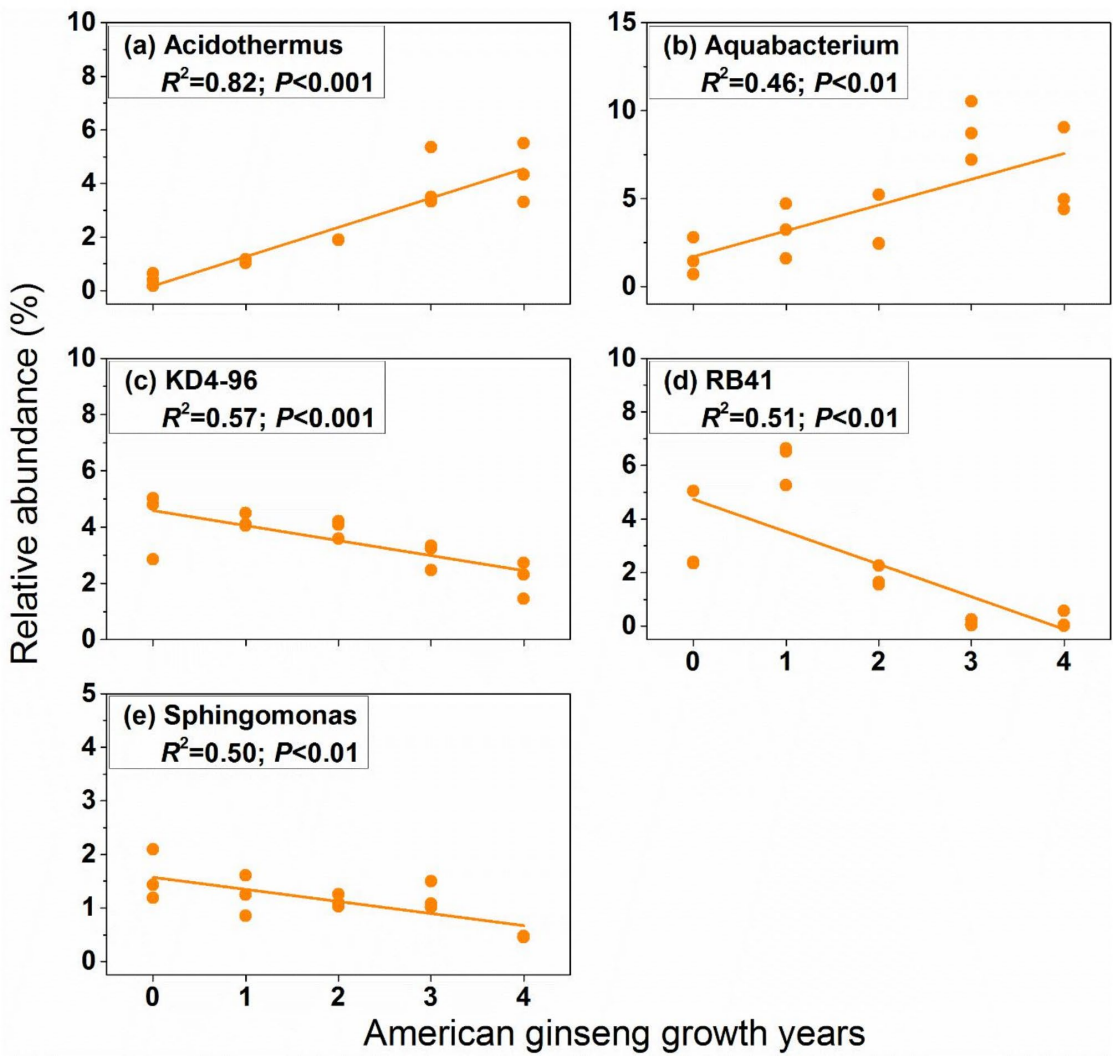
The relationship between the relative abundance of dominant bacterial genus (mean relative abundance > 1%) and American ginseng growth years. For each response variable, regression was estimated using a linear model with years of American ginseng cultivation as a continuous predictor. *R*^*2*^ is coefficient of determination; *P* is significance of the regression. Only these phyla significantly differed in different sampling times are shown

**Fig. 6:**
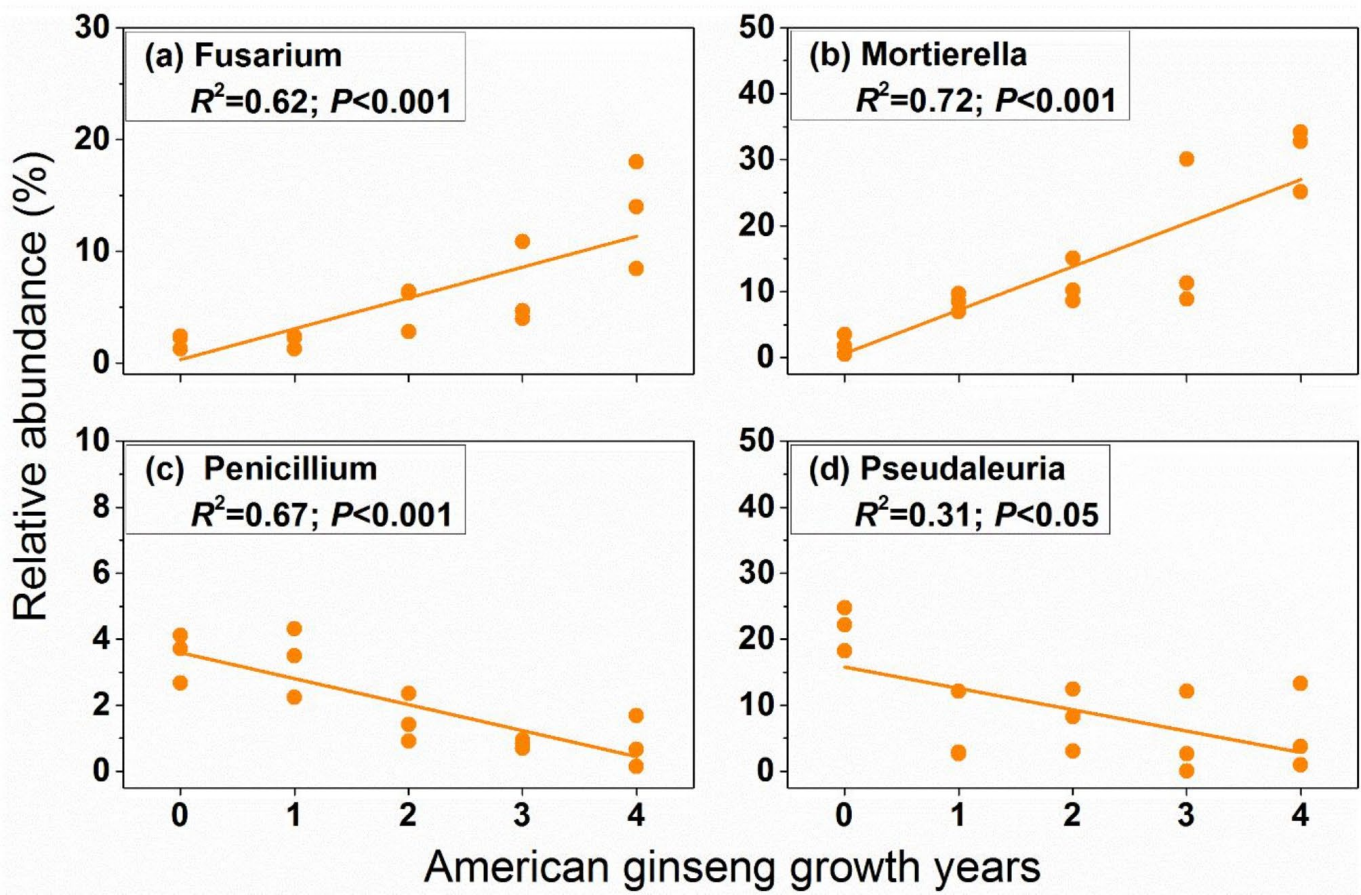
The relationship between the relative abundance of dominant fungal genus (mean relative abundance > 1%) and American ginseng growth years. For each response variable, regression was estimated using a linear model with years of American ginseng cultivation as a continuous predictor. *R*^*2*^ is coefficient of determination; *P* is significance of the regression. Only these genera significantly differed in different sampling times are shown

At the genus level, a linear regression analysis was used to calculate the relationship between the relative abundance of dominant bacterial and fungal genus (mean relative abundance > 1%) and American ginseng growth years. The results revealed that relative abundance of bacterial *Aquabacterium* and *Acidothermus* increased with the American ginseng growth years, while the relative abundance of *KD4-96*, *RB41* and *Sphingomonas* decreased (Fig. 5). At fungal genus level, the relative abundance of *Fusarium* and *Mortierella* showed positive relationship with the years of American ginseng planted, and the relative abundance of *Penicillium* and *Pseudaleuria* showed negative relationship with the years of American ginseng planted (Fig. 6).

**Fig. 7:**
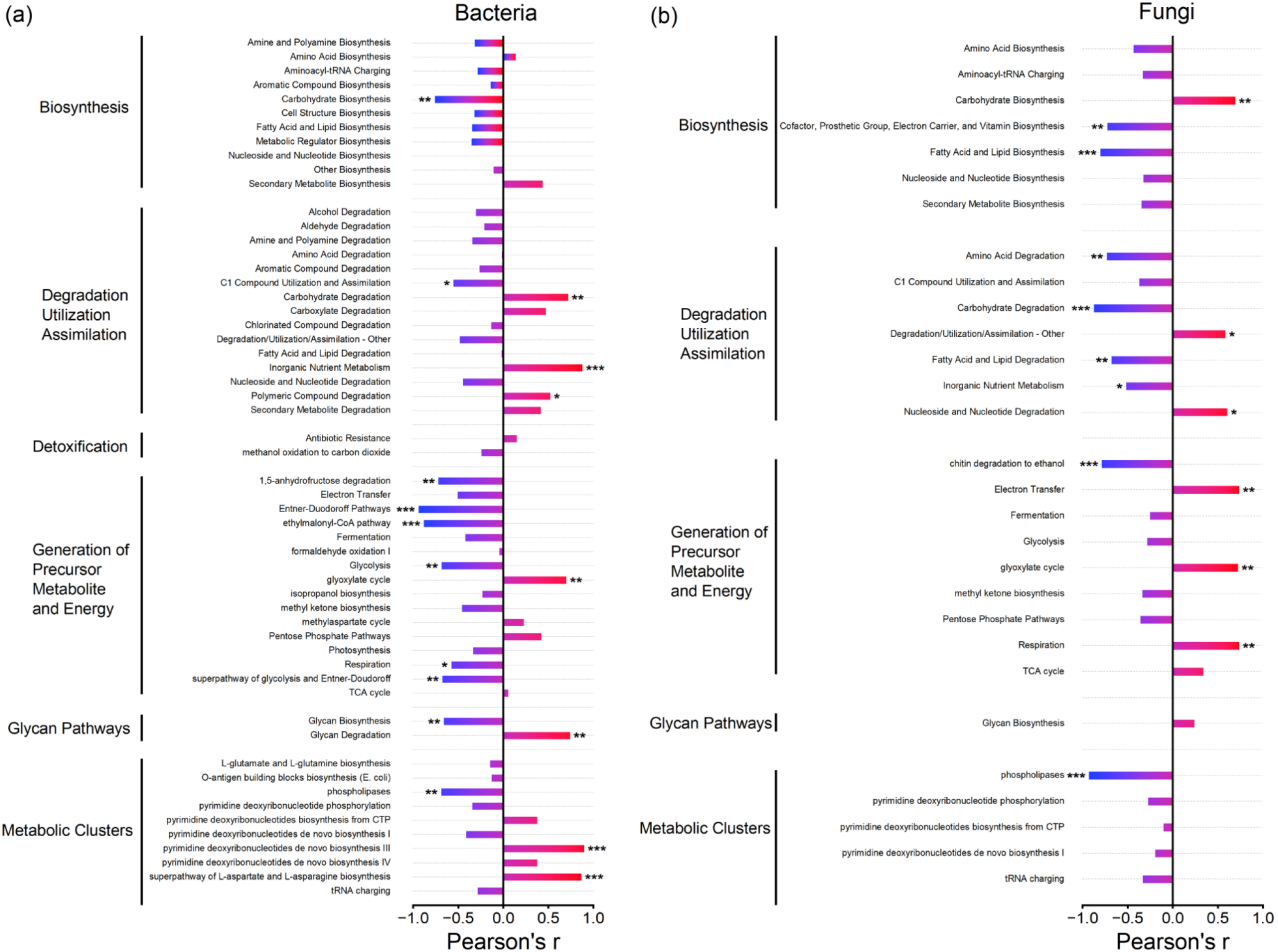
Responses of the abundances of metabolic pathways from general bacteria (**a**) and fungi (**b**) predicted using PICRUSt2 to the planting years of American ginseng. For each response variable, regression was estimated using a linear model with years of American ginseng cultivation as a continuous predictor. Bars represent the correlation coefficients (Pearson’s r). * *P* < 0.05; ** *P* < 0.01; *** *P* < 0.001

The PICRUSt2 was used to predict the abundances of different metabolic pathways based on 16 S rRNA gene and 18 S rRNA gene sequencing data. For bacteria, planting American ginseng abundantly enriched the pathways of carbohydrate degradation, inorganic nutrient metabolism, polymeric compound degradation, glyoxylate cycle, glycan degradation, pyrimidine deoxyribonucleotides de novo biosynthesis III and superpathway of L-aspartate and L-asparagine biosynthesis, and significantly weakened the pathways of carbohydrate biosynthesis, C1 compound utilization and assimilation, 1,5-anhydrofructose degradation, Entner-Duodoroff pathways, ethylmalonyl-CoA pathway, glycolysis, respiration, superpathway of glycolysis and Entner-Doudoroff, glycan biosynthesis and phospholipases (Fig. 7a). On the other hand, for fungi, with the increase of planting years of American ginseng, the pathways of carbohydrate biosynthesis, degradation/utilization/assimilation-other, nucleoside and nucleotide degradation, electron transfer, glyoxylate cycle and respiration was abundantly enriched, while the pathways of cofactor, prosthetic group, electron carrier and vitamin biosynthesis, fatty acid and lipid biosynthesis, amino acid degradation, carbohydrate degradation, fatty acid and lipid degradation, inorganic nutrient metabolism, chitin degradation to ethanol and phospholipases was notably weakened (Fig. 7b).

The RDA results revealed that soil pH and available potassium observably affected soil bacterial community and functional composition. For both bacteria and fungi, soil pH, with highest explanations, was the main factor to influence their community composition and function (Table 2).

**Table 2:** Results from the RDA (redundancy analysis) of soil bacterial and fungal community composition and functional composition with soil properties

	Soil properties	Explains (%)	*P*
Bacterial community composition	pH	21.9	< 0.01
	Available potassium	13.8	< 0.01
	Inorganic N	6.4	0.684
Bacterial functional composition	pH	28.7	< 0.01
	Available potassium	17.9	< 0.01
	Inorganic N	9.2	0.216
Fungal community composition	pH	12.4	< 0.01
	Available potassium	9.8	< 0.05
	Inorganic N	7.8	0.028
Fungal functional composition	pH	41.2	< 0.01
	Available potassium	22.0	< 0.05
	Inorganic N	2.1	0.950

The Pearson correlation analysis was q performed to determine the correlation between soil physicochemical properties and these genera which relative abundance significantly changed with continuous planting American ginseng. The results showed that the relative abundance of all dominant bacterial and fungal genera were significantly correlated with soil pH, either positively or negatively (Table 3). For bacteria, the relative abundance of *KD4-96*, *RB41* and *Sphingomonas* were negatively correlated with soil available potassium. For fungi, the relative abundance of *Fusarium* and *Mortierella* showed positive relationship with soil available potassium, and the relative abundance of *Penicillium* and *Pseudaleuria* showed negative relationship with soil available potassium (Table 3).

**Table 3:** Relationship between soil pH, soil available potassium, soil inorganic N and the relative abundance of dominant bacterial and fungal genera from a linear regression analysis

Taxa		pH	Available potassium	Inorganic *N*
Bacteria	Acidothermus	-0.83***	0.46	-0.01
	Aquabacterium	-0.68**	0.39	-0.06
	KD4-96	0.65**	-0.59*	-0.09
	RB41	0.67**	-0.66**	0.11
	Sphingomonas	0.69**	-0.61*	-0.21
Fungi	Fusarium	-0.70**	0.57*	0.05
	Mortierella	-0.79***	0.78***	0.34
	Penicillium	0.83***	-0.72**	-0.05
	Pseudaleuria	0.66**	-0.36	-0.19

Only these genera which relative abundance significantly changed with continuous planting American ginseng are included in the linear regression analysis. The correlation coefficients (Pearson’s *r*) are presented with the level of significance. * *P* < 0.05; ** *P* < 0.01; *** *P* < 0.001

## Discussion

The results indicate that planting American ginseng had strong negative influences on bacterial and fungal alpha diversity, and the longer the planting years, the more diversity decreased. The main reason may be that the cultivation of American ginseng led to soil acidification, as this study has shown that soil pH value decreased with increasing planting years. It is well-known that soil pH is the main factor affecting the diversity of soil microbial communities in different ecosystems [27–30]. The effects of soil pH on soil microorganisms can be divided into direct effects and indirect effects. Soil pH can directly affect soil microbial communities, because most soil microbial groups have an optimal pH range for growth. Once soil pH exceeds this range, microbial growth will be limited and therefore microbial diversity will be reduced [29, 31]. The indirect effects mean that soil acidification can adversely affect the growth of soil bacterial communities by increasing the solubility of some toxic metals (like Al, Fe, and Cu) in soil [28, 32]. Since the bacteria were more sensitive to pH changes, the diversity of bacterial community decreased significantly when soil pH decreased after the planting of American ginseng. Consequently, planting American ginseng by decreasing soil pH reduced the soil bacterial and fungal alpha diversity.

In this research, planting American ginseng distinctly altered the soil bacterial and fungal community composition and functional composition, which may be an important reason for continuous cropping obstacle. For bacterial communities, our results revealed that the relative abundance of *Acidothermus* largely increased with the American ginseng growth years. Bacteria identified into this genus are acidophilic and thermophilic, and capable of producing many thermostable cellulolytic enzymes [33, 34]. As soil pH value decreasing with growth years, *Acidothermus* could outcompete in the bacterial warfare and gradually became the dominant genus in soil bacterial communities.

There ae some bacterial taxa that showed negative relationship between relative abundance with growth year, especially for *KD4-96* (Chloroflexi), *RB41* (Acidobacteria) and *Sphingomonas.* Most of these bacteria are beneficial for soil health. For example, *RB41* plays an important role in maintaining soil biogeochemical cycling under long-term high pollution stress [35, 36]. Study on the rhizosphere soil of *Fritillaria ussuriensis* with different health levels showed that the *RB41* genus in the healthy soil had the highest relative abundance [37]. *Sphingomonas* from Proteobacteria can degrade some toxic substances in soil, such as diphenylether, atrazine and phenylurea herbicide isoproturon, and promote crop resistance to a variety of pathogens [1, 38]. Furthermore, it was found that *KD4-96* was a kind of heavy metal-resistant bacterium, which played a key role in the biotransformation process of heavy metals [39, 40]. Moreover, genus *KD4-96* can be largely affected by soil properties [41]. The decrease of relative abundance of *KD4-96* might be due to the changes in soil pH, since soil pH has been regarded as the main driving factor accounting for variation in diversity and functions of microbial community [42].

Pathogenic fungi, like *Fusarium*, were enriched with planting American ginseng. *Fusarium* can infect many plant species and causing root rot and wilt diseases [43]. Some *Fusarium* strains have been isolated from the infected roots of American ginseng, including *Fusarium solani*, *F. oxysporum*, *F. avenaceum*, and *F. equiseti* [44]. Bi et al. [45] further confirmed that *F. solani* and *F. oxysporum* were the main pathogens causing root rot. The enrichment of *Fusarium* might contribute to the continuous cropping obstacles and decrease of production, which need further concern.

In this study, *Mortierella* was the most abundant fungal genus. Many plants growth-promoting fungi are involved in this genus. Previous studies have confirmed that *Mortierella* can be isolated from the roots of red-skin Asian ginseng (*P. ginseng*) [46]. Here, it was suggested that the relative abundance of *Mortierella* increases with the years of American ginseng cultivation, and that even reached more than 30% in the fourth year of planting ginseng. This indicated that soil microbial community is resilient to the increase of pathogens and the changed soil pH. However, knowledge about interactions between pathogenic fungi and plants growth-promoting fungi are still limited, especially for that between *Fusarium* and *Mortierella*. Therefore, future studies should focus on deciphering the intergeneric microbial interactions to better understand the impacts of continuous cropping on American ginseng plantation.

## Conclusion

In this research, the alpha diversity of bacteria and fungi decreased with the increase of American ginseng planting years. American ginseng culture also significantly altered the community and functional composition of soil bacteria and fungi. Particularly, the relative abundance of some beneficial soil microbes, such as *KD4-96*, *RB41* and *Sphingomonas*, gradually decreased with the increase of American ginseng planting years. On the other hand, after planting American ginseng, the relative abundance of some pathogenic fungi, such as *Fusarium* significantly increased. In addition, the cultivation of American ginseng resulted in soil acidification, which was significantly associated with the decrease of soil microbial diversity and changes in community and functional composition after American ginseng cultivation, suggesting that soil pH was a key environmental factor affecting soil microbial communities in American ginseng cultivation farmland. In summary, our results indicated that soil acidification and the changes of bacterial and fungal communities maybe the main reason caused replanting problems of American ginseng. This study contributes to an improved understanding of the replanting problems of American ginseng and provide a theoretical basis for soil improvement after the harvest of American ginseng.

## Data Availability

Sequence data that support the findings of this study have been deposited in the NCBI BioProject repository with the BioProject ID: PRJNA1177235 and PRJNA1177246.
